# Integrating plan complexity and dosiomics features with deep learning in patient-specific quality assurance for volumetric modulated arc therapy

**DOI:** 10.1186/s13014-023-02311-7

**Published:** 2023-07-11

**Authors:** Ce Han, Ji Zhang, Bing Yu, Haoze Zheng, Yibo Wu, Zhixi Lin, Boda Ning, Jinling Yi, Congying Xie, Xiance Jin

**Affiliations:** 1 Department of Radiotherapy Center, 1st Affiliated Hospital of Wenzhou Medical University, Wenzhou, China; 2 Department of Medical and Radiation Oncology, 2nd Affiliated Hospital of Wenzhou Medical University, Wenzhou, China; 3 School of Basic Medical Science, Wenzhou Medical University, Wenzhou, China

**Keywords:** Deep learning, Dosiomics feature, Plan complexity, Classification, Patient-specific quality assurance

## Abstract

**Purpose:**

To investigate the feasibility and performance of deep learning (DL) models combined with plan complexity (PC) and dosiomics features in the patient-specific quality assurance (PSQA) for patients underwent volumetric modulated arc therapy (VMAT).

**Methods:**

Total of 201 VMAT plans with measured PSQA results were retrospectively enrolled and divided into training and testing sets randomly at 7:3. PC metrics were calculated using house-built algorithm based on Matlab. Dosiomics features were extracted and selected using Random Forest (RF) from planning target volume (PTV) and overlap regions with 3D dose distributions. The top 50 dosiomics and 5 PC features were selected based on feature importance screening. A DL DenseNet was adapted and trained for the PSQA prediction.

**Results:**

The measured average gamma passing rate (GPR) of these VMAT plans was 97.94% ± 1.87%, 94.33% ± 3.22%, and 87.27% ± 4.81% at the criteria of 3%/3 mm, 3%/2 mm, and 2%/2 mm, respectively. Models with PC features alone demonstrated the lowest area under curve (AUC). The AUC and sensitivity of PC and dosiomics (D) combined model at 2%/2 mm were 0.915 and 0.833, respectively. The AUCs of DL models were improved from 0.943, 0.849, 0.841 to 0.948, 0.890, 0.942 in the combined models (PC + D + DL) at 3%/3 mm, 3%/2 mm and 2%/2 mm, respectively. A best AUC of 0.942 with a sensitivity, specificity and accuracy of 100%, 81.8%, and 83.6% was achieved with combined model (PC + D + DL) at 2%/2 mm.

**Conclusions:**

Integrating DL with dosiomics and PC metrics is promising in the prediction of GPRs in PSQA for patients underwent VMAT.

## Introduction

Due to the inverse nature of intensity-modulated radiotherapy (IMRT) and volumetric modulated arc therapy (VMAT) planning, patients-specific quality assurance (PSQA) is an imperative step to detect potential errors resulted from an inaccurate dose calculation, a failure of the record-and-verify system, or delivery errors in the linear accelerator to ensure the accuracy of IMRT/VMAT delivery [1, 2]. Typically, PSQA is performed by measuring the radiation dose of IMRT/VMAT plans with 2D or 3D diode arrays and then comparing measured dosimetric distribution with planned one using a gamma passing rate (GPR). However, this traditional PSQA increases the overall clinical workload and usage of resources [3, 4]. Traditional PSQA also hinders the application of online adaptive radiotherapy, which requires a fast real time treatment planning and QA process [5, 6].

More sophisticated independent 3D dose calculation algorithms, such as convolution-superposition or Monte Carlo were introduced to verify the IMRT/VMAT plans virtually [7, 8]. On the other hand, studies demonstrated that treatment plan complexity (PC) and Linac performance metrics will influence the radiation therapy delivery [9]. PC metrics of modulation complexity score (MCS), leaf motion constraints, average leaf travel (LT), MCS applied to VMAT (MCSv), etc., had been investigated to assess the relation between overall PC and PSQA results [10, 11]. With the emerging and application of machine learning (ML) and deep learning (DL), more straightforward, less resource-intensive, efficient PSQA methods using treatment PC metrics and/or linac performance metrics were proposed to predicted the GPRs directly [12–15]. However, only weak correlations were reported between passing rates and these metrics as different aspects of the complexity of the plans might interact each other and associate with the failing of PSQA [16, 17].

Recently, radiomics with quantitative extracted image features had been applied to predict simulated radiotherapy errors for PSQA [18]. Gamma images resulted from IMRT plannar dose QA were evaluated to classify the presence or absence of introduced radiotherapy treatment delivery errors with convolutional neural networks (CNN), which indicates radiomic quality assurance is a promising direction for clinical radiotherapy [19]. Radiomics features extracted from dosimetric distribution (dosiomics) had been suggested to combine with PC metrics to improve the prediction and classification performance for GPR with ML [20]. Studies demonstrated that combining DL with radiomics through information fusion is able to improve the prediction ability of models [21, 22]. The purpose of this study is to investigate the feasibility and performance of DL integrated with dosiomics and PC features in the PSQA for patients underwent VMAT.

## Materials and methods

### Study design

**Fig. 1:**
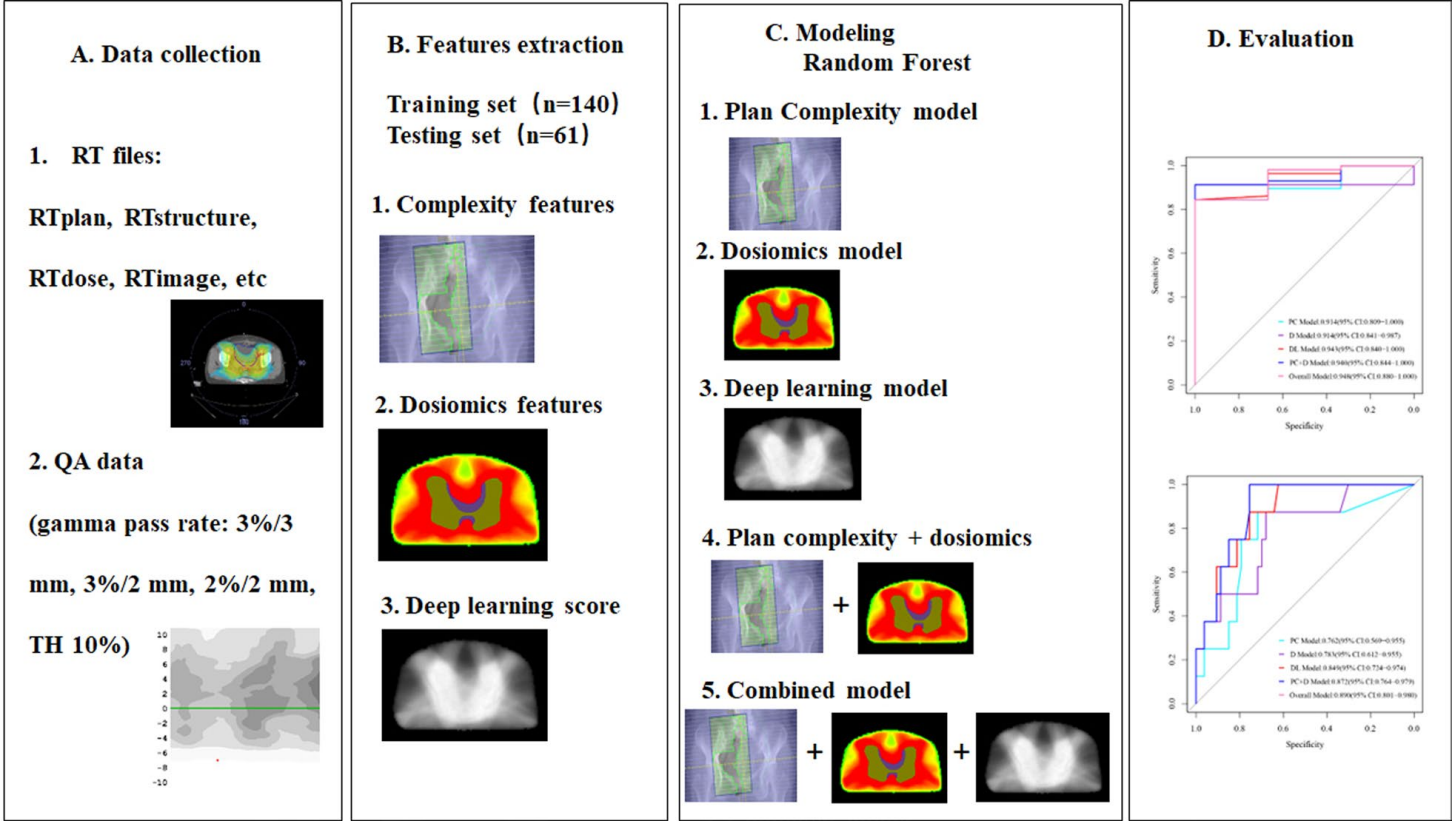
The flowchart for the overall study design

Figure 1 shows the flowchart for the overall study design, which consists of four-steps: (A) collection of the radiotherapy (RT) files, including RTplan, RTdose, RTstructure and RTimages, and corresponding PSQA data from each VMAT plan; (B) extraction of the complexity features of the plans, 3D dosiomics features of planning target volume (PTV) and overlapping region; (C) feature selection and modeling; (D) model evaluation and comparison.

### Patients and PSQA data

Patients underwent two-arc VMAT with measured PSQA results were retrospectively reviewed and enrolled in this study. VMAT plans were generated by commercial treatment planning system (TPS) (Monaco 5.1.1; Elekta, Crawley, UK) for a 6-MV X-ray beam with a dose grid size of 3.0 × 3.0 mm. Detailed optimization parameters and procedures had been reported previously [23, 24]. The PSQA measurements were conducted using a 3D diode array ArcCHECK (Model 1220) and SNC Patient (v.6.2.1; Sun Nuclear Corporation) with Elekta Synergy linac (Elekta Ltd, Crawley, UK), which was equipped with an 80-leaf multileaf collimator (MLCi2TM, Elekta Ltd, Crawley, UK). GPRs of three different acceptance criteria: 3%/3 mm, 3%/2 mm and 2%/2 mm with a 10% lower dose threshold were calculated and recorded [25, 26].

### Complexity metrics and dosiomics features

House-built algorithm based on Matlab 2016a (Mathwork Inc., USA) was built to read and calculated complexity metrics from exported DICOM-RT files from TPS, which includes RTplan, RTstructure, RTdose, RTimage etc. A total of 13 PC metrics were calculated, which includes monitor units (MUs), MU per control point (MU/CP), the proportion of CPs with MU < 3 (%MU/CP < 3), small segment area per CP (SA/CP), the percentage of CPs with segment area < 5 × 5 cm^2^ (%SA < 5 × 5 cm^2^), modulation complexity score of VMAT per arc (MCSv/Arc), leaf travel (LT) distance, Gantry spacing, etc., as reported in a previous study [11].

**Fig. 2:**
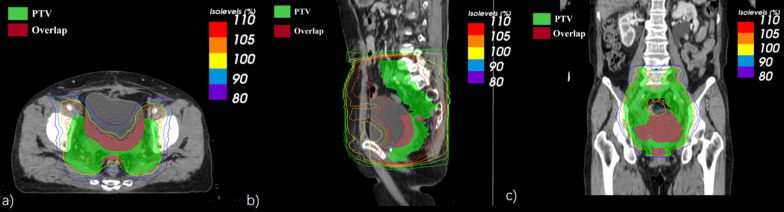
A typical PTV and overlap regions of a cervical cancer patient with 3D dose distribution. **a** horizontal; **b** sagittal; **c** coronal

Dosiomics features were extracted from PTV and overlap regions (PTV overlapped with organs at risk) with 3D dose distributions using the PyRadiomics package (version 2.1.2) of Python (version 3.8) [20]. Figure 2 shows a typical PTV and overlap regions of a cervical cancer patient with 3D dose distribution for radiomics feature extraction, which contains the shape, image statistical values, and heterogeneity of the dose distribution. All the dose images were resampled to a pixel spacing of 1 × 1 × 1mm^3^ with B-spline interpolation algorithm to standardized feature computation. The pixel values were discretized into equally spaced bins using a fixed bin width of 25 Hounsfield Units to eliminate the influence of different grayscale ranges and ensure better comparability. A total of 833 features were extracted, which includes105 first-order features, and 728 s- and higher-order features of shape, gray level co-occurrence matrix (GLCM), gray level run length matrix (GLRLM), gray level size zone matrix (GLSZM), gray level dependence matrix (GLDM) and neighboring gray tone difference matrix (NGTDM) according to the image biomarker standardization initiative (IBSI) reporting guidelines [27].

### Feature selection and modeling

The data was randomly divided into a training and testing set at a ratio of 7:3. The Random Forest (RF) algorithm was applied to select the top 50 dosiomics features based on the mean decrease accuracy in the training set [28]. Then a total of 50 dosiomics features from the PTV and overlap regions and 5 complexity features were selected in the training cohort according to the feature importance screening and applied for the construction of signature via RF. During modeling, a GPR higher than 95% at 3%/3 mm, higher than 90% at 3%/2 mm, or higher than 80% at 2%/2 mm was set as the action limit of “pass”, otherwise, it was “fail”, respectively [29].

### Deep learning model

**Fig. 3:**
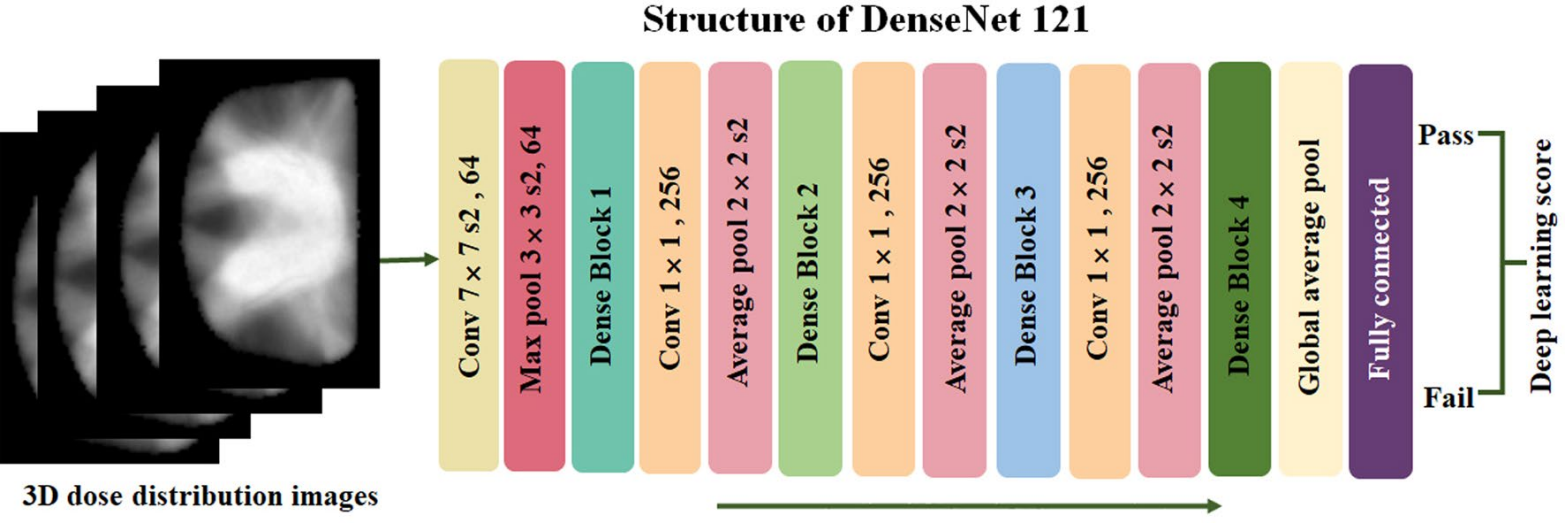
Framework for PSQA prediction based on DenseNet 121 training

In the preprocessing, 3D dose distribution data of PTV and overlap were converted to images of NRRD format and normalized to 96 × 96 × 96. A DenseNet 121 was adapted and trained for the PSQA prediction in the Medical Open Network for Artificial Intelligence (MONAI), an open-source framework for DL in medical imaging based on Pytorch. There are four dense blocks in the DenseNet 121. The layer between two adjacent blocks is called the transition layer, which changes the feature map size by convolution and pooling. DL models were trained for at least 200 epochs using the Adam optimizer and a learning rate of 0.00001. Models were trained from scratch with no pre-training with their last classifier layer would be a sigmoid layer capable of performing binary classification. To prevent overfitting and improve the generalization of the models, different data enhancement methods were applied during training. The DL score of the models with best performance in the training set will combined with dosiomics signature in the RF model for final prediction, as shown in Fig. 3.

### Model evaluation and statistical analysis

A total of five models were generated and compared, namely, PC models (based on PC metrics), dosiomics models (D), DL models, PC + D models (combined PC metrics and dosiomics features), and overall model (DL + PC + D, integrating DL with PC metrics and dosiomics features). The receiver operating characteristic (ROC) curves and the area under curve (AUC) were applied for the evaluation and comparison of these models using “pROC” package of R analysis platform (version 3.0.1, MathSoft). RF algorithm was based on “randomForest” package. The classification algorithm was based in part on MONAI (version 0.8.1) and other open-source projects available at https://github.com/Project-MONAI/tutorials. Other data analysis was performed using Python 3.6.0 and custom-written software in MATLAB R2016a. For all tests, *p* < 0.05 was considered as statically significant.

## Results

A total of 201 two-arc VMAT plans were enrolled in this study with 135 pelvis plans and 66 head and neck (H&N) plans at a prescription dose to PTV of 45 Gy (1.8 Gy/fractions) and 60 Gy (2.0 Gy/ fractions), respectively. Pelvis plans includes gynecologic, rectal, and prostate cancer patients. H&N plans includes nasopharyngeal carcinoma, laryngeal carcinoma, and hypopharyngeal carcinoma. Detailed characteristics of these plans were summarized in Table 1.

**Table 1:** The characteristics of patients enrolled in this study

Disease site	Case number	%	Prescription
Pelvis	135	67.16	45 Gy/25fx
Gynecologic cancer	83	41.29	
Rectal cancer	44	21.89	
Prostate cancer	8	3.98	
Head and Neck	66	32.84	60 Gy/30fx
Nasopharyngeal cancer	34	16.95	
Laryngeal cancer	19	9.45	
Hypopharyngeal cancer	13	6.47	

**Fig. 4:**
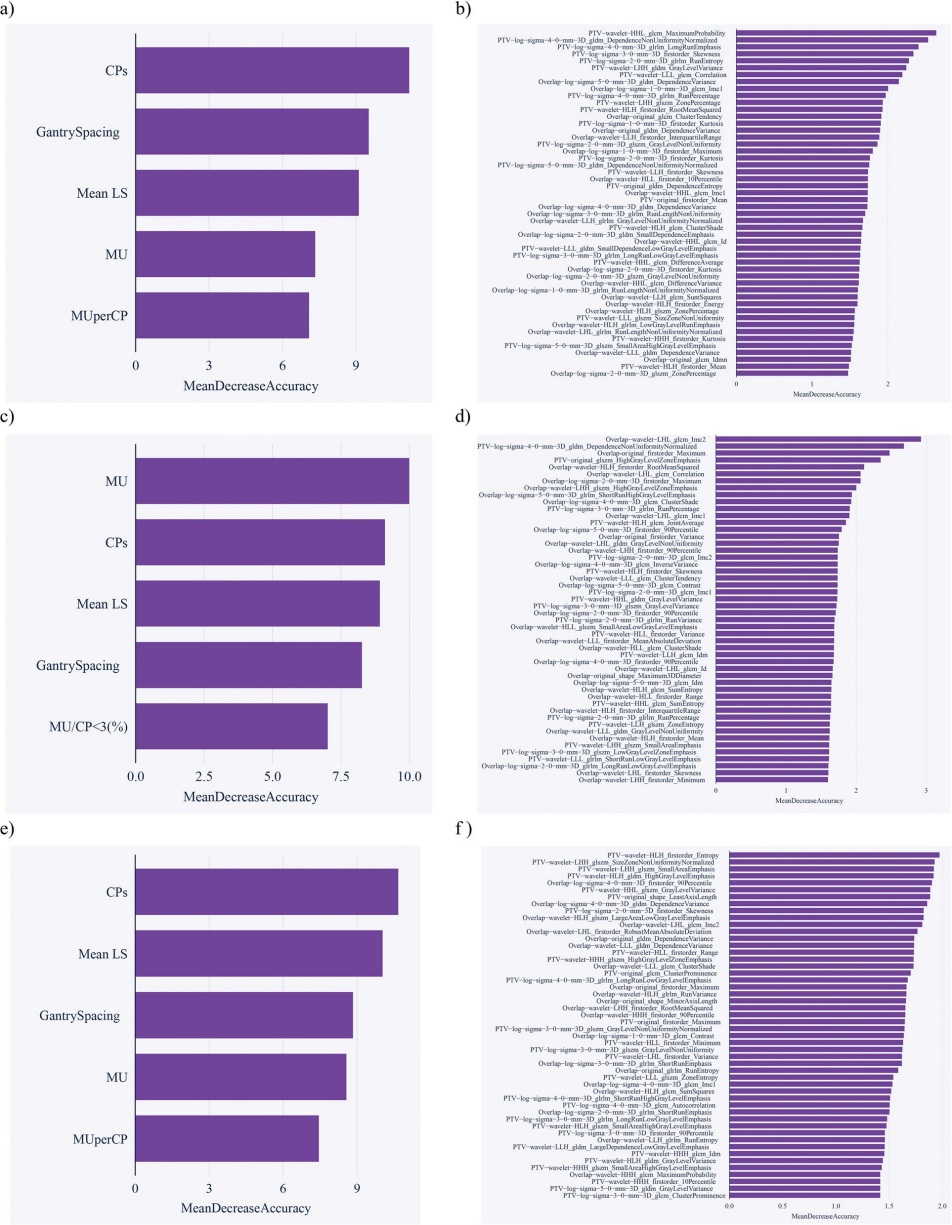
According to the important value of RF method, the top 5 complexity features and 50 dosiomics features were selected for GPR prediction with different PSQA criteria. **a** the top 5 complexity features of 3%/3 mm; **b** the top 50 dosiomics features of 3%/3 mm; **c** the top 5 complexity features of 3%/2 mm; **d** the top 50 dosiomics features of 3%/2 mm; **e** the top 5 complexity features of 2%/2 mm; **f** the top 50 dosiomics features of 2%/2 mm

Table 2 shows collected PSQA results of these VMAT plans with an average GPS of 97.94% ± 1.87%, 94.33% ± 3.22%, and 87.27% ± 4.81% under the criteria of 3%/3 mm, 3%/2 mm, and 2%/2 mm, respectively. According to the important value of RF method, the top 5 complexity features and 50 dosiomics features were selected for GPR prediction with different PSQA criteria of 3%/3 mm, 3%/2 mm and 2%/2 mm, as shown in Fig. 4.

**Table 2:** Recorded results of patient-specific quality assurance

Measured GPR	3%/3 mm	3%/2 mm	2%/2 mm
Mean (%)	97.94	94.33	87.27
SD (%)	1.87	3.22	4.81
Case numbers			
GPR ≥ 95%	186	106	7
90% ≤ GPR < 95%	15	75	58
80% ≤ GPR < 90%	0	22	115
GPR < 80%	0	0	21

**Fig. 5:**
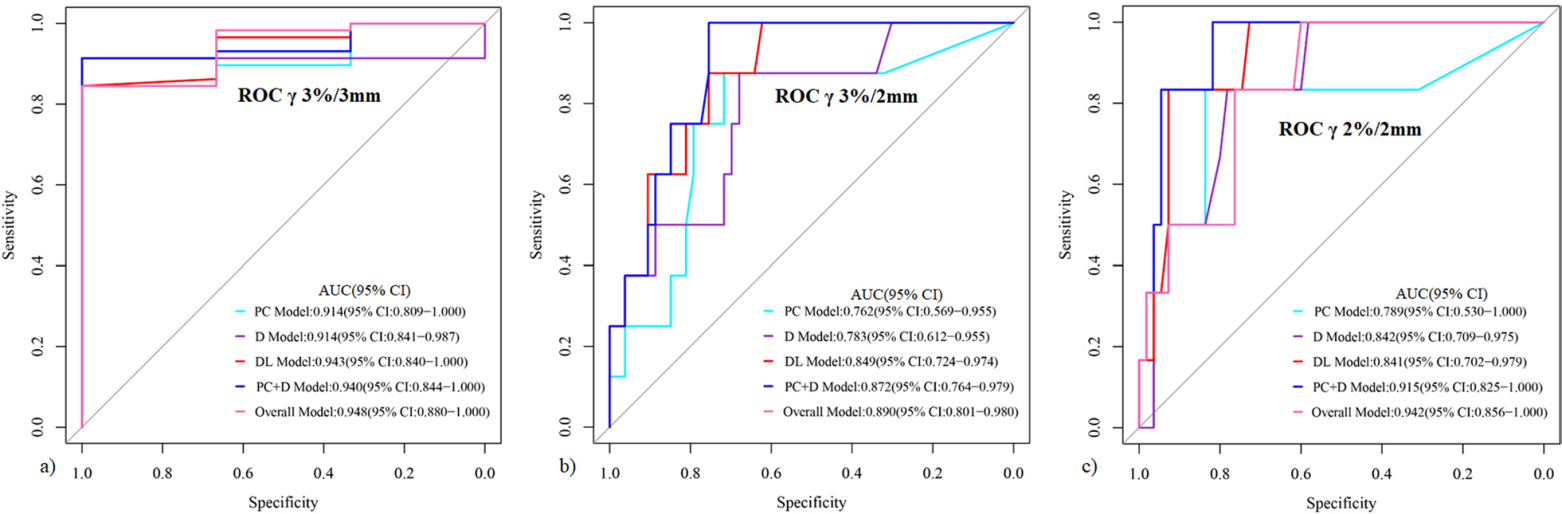
Performance of prediction models with plan complexity (PC), dosiomics features (D), deep learning (DP) in the testing set. **a** Prediction models of 3%/3 mm; **b** Prediction models of 3%/2 mm; **c** Prediction models of 2%/2 mm

Figure 5 shows the performance of PC, D, DL, PC + D, and overall model for the PSQA criteria of 3%/3 mm, 3%/2 mm, and 2%/2 mm, respectively. The overall model achieved a best AUC of 0.948(95% CI 0.880–1), 0.890(95% CI 0.801–0.980) and 0.942(95% CI 0.856–1) at 3%/3 mm, 3%/2 mm, and 2%/2 mm, respectively. Detailed performance of these models was presented in Table 3.

**Table 3:** Performance of different models for the prediction of patient-specific quality assurance with different percent dose difference/distance to agreement criteria

Criteria	Models/parameters	AUC (95% CI)	Sensitivity	Specificity	Accuracy
3%/3 mm	PC model	0.914 (0.809–1.000)	0.845	1.000	0.869
	D model	0.914 (0.841–0.987)	0.914	1.000	0.918
	DL model	0.943 (0.840–1.000)	0.845	1.000	0.853
	PC + D model	0.940 (0.844–1.000)	0.845	1.000	0.853
	Overall model	0.948 (0.880–1.000)	0.914	1.000	0.918
3%/2 mm	PC model	0.762 (0.569–0.955)	0.875	0.717	0.738
	D model	0.783 (0.612–0.955)	0.875	0.679	0.705
	DL model	0.849 (0.724–0.974)	1.000	0.566	0.623
	PC + D model	0.872 (0.764–0.979)	0.875	0.755	0.771
	Overall model	0.890 (0.801–0.980)	1.000	0.755	0.787
2%/2 mm	PC model	0.789 (0.530–1.000)	0.833	0.836	0.836
	D model	0.842 (0.709–0.975)	0.833	0.782	0.787
	DL model	0.841 (0.702–0.979)	1.000	0.600	0.639
	PC + D model	0.915 (0.825–1.000)	0.833	0.927	0.918
	Overall model	0.942 (0.856–1.000)	1.000	0.818	0.836

*PC* plan complexity, *D* dosiomics, *DL* deep learning, *AUC* area under curve

## Discussion

In this study, the feasibility of combining DL with dosiomics features and PC metrics for the PSQA of patients underwent VMAT were investigated. A best AUC of 0.942 with a sensitivity, specificity and accuracy of 100%, 81.8%, and 83.6% was achieved with combined overall model at the criteria of 2%/2 mm.

PSQA is an imperative step to assess the reliability of treatment delivery of IMRT/VMAT plans and to improve the patient safety due to the increased dosimetric uncertainty resulted from inverse planning [29]. Although many studied questioned the clinical significance of GPR, gamma analysis is still the most widely applied PSQA methods [30]. GPR with the criterion of 3%/3 mm is commonly recommended and routinely applied in clinical practice for IMRT/VMAT PSQA [29, 31]. Previous studies suggested that different criteria of GPR should be applied to detect different types of errors during IMRT/VMAT delivery [32, 33]. Therefore, criteria of 3%/3 mm, 3%/2 mm, and 2%/2 mm were applied in this study for the assessment of PSQA. It was consistent with previous study, the GPR was decreased from 97.94 to 87.27% with the increased strict criteria from 3%3 mm to 2%/2 mm.

Virtual PSQA without actual measurement is desirable in the treatment planning process as to identify failing plans early in the process and to increase the efficiency of the PSQA practice [34]. Studies generally agreed that the GPRs of IMRT/VMAT plans are heavily contingent on PC [16, 35]. In this study, virtual prediction models based on complexity metrics demonstrated that for GPRs with different criteria, the associated complex metrics were different. As shown in Fig. 4, the complexity metrics and their corresponding weights for GPRs of 3%/3 mm, 3%/2 mm and 2%/2 mm were CPs, Gantry spacing, Mean LT, MU and MU/CP; MU, CPs, Mean LT, Gantry Spacing, MU/CP < 3%; and CPs, Mean LT, Gantry Spacing, MU, MU/CP, respectively. Similarly, different complexity metrics were reported in different studies for the prediction of GPRs. Valdes et al. reported that MU/Gy, small aperture score, irregularity factor, and fraction of the plan delivered at the corners of a 40 × 40 cm field were the most important metrics that determines the GPRs at 3%/3 mm [12]. Shen et al. demonstrated for patients with nasopharyngeal cancer underwent two-arc VMAT treatment, complexity metrics of MU/CP and segment area (SA) per control point (SA/CP) were highly correlated with GPRs [11]. As shown in Table 3, the models based on complexity metrics demonstrated the lowest AUC for the GPRs prediction. It was consistent with previous studies that the correlations between complexity metrics and GPRs are generally weak [36, 37].

In this study, with the application of dosiomics, radiomics features extracted from dosimetric distributions, an accuracy of 91.8%, 70.5% and 78.7% was achieved in the prediction of GPRs at the criteria of 3%/3 mm, 3%/2 mm and 2%/2 mm, respectively. This is better than the reported maximum accuracy of 77.3% in the study of Nyflot et al., in which radiomics based on planar dose maps was applied for IMRT PSQA at 3%/3 mm [19]. In the prostate QA gamma deep learning prediction model, the input training data also include PTV and overlapping regions, suggesting that these areas had some important information.^15^ In this study, the AUC of dosiomics features extracted from the PTV and overlapping region at 3%/2 mm, 2%/2 mm were 0.783 and 0.842, respectively, which is superior to the reported dosiomics AUCs of 0.78 and 0.81 in the study of Hirashima et al. [20]. The AUC and sensitivity of combined model with complexity features and dosiomics features at 2%/2 mm were 0.915 and 0.833, respectively, which is also comparable to the reported 0.83 and 0.90 in the study of Hirashima, et al.

Handcrafted features, such as radiomics and dosiomics, were generally the main approaches for medical imaging analysis. With the development of DL, studies indicated that combining the DL models with the handcrafted features with learned knowledge may improve the performance of these deep learning models [38, 39], In this study, the AUCs of DL models were improved from 0.943, 0.849, 0.841 to 0.948, 0.890, 0.942 in the combined overall models at the GPR criteria of 3%/3 mm, 3%/2 mm and 2%/2 mm, respectively. This is also indicated the improvement of adding DL for automatic PSQA in comparison with using only PC and dosiomics features in that of Hirashima et al. [20]. For the criteria of 3%/3 mm with a relatively high GPRs, the combined overall model did not show much improvement.

The cases enrolled in this study for PSQA were patients with gynecologic cancer, rectal cancer, prostate cancer and head-and-neck cancer. VMAT plans with different prescription doses were investigated. To further generalize the application of these models, VMAT plans for other site of cancer, such as esophageal, lung, and breast cancer, and from multiple institutions should be included in our future study. The reliability of GPRs is questioned, additional evaluation indices, such as individual volume-based gamma index, DVH based metrics should be further investigated in our future study.

## Conclusions

Dosiomics features were feasible for the PSQA of VMAT. Integrating DL with dosiomics and PC metrics is promising in the prediction of GPRs in PSQA for patients underwent VMAT.

## Data Availability

Research data are stored in an institutional repository and will be shared upon request to the corresponding author.

## Abbreviations

DL: Deep learning
PC: Plan complexity
PSQA: Patient-specific quality assurance
VMAT: Volumetric modulated arc therapy
RF: Random forest
PTV: Planning target volume
GPR: Gamma passing rate
AUC: Area under curve
IMRT: Intensity-modulated radiotherapy
MCS: Modulation complexity score
LT: Leaf travel
ML: Machine learning
CNN: Convolutional neural networks
TPS: Treatment planning system
MUs: Monitor units
